# Evaluation of bony features associated with hip instability in hip dysplasia

**DOI:** 10.1007/s00590-025-04336-y

**Published:** 2025-05-20

**Authors:** Takeshi Shoji, Hideki Shozen, Shinichi Ueki, Hiroki Kaneta, Hiroyuki Morita, Yosuke Kozuma, Nobuo Adachi

**Affiliations:** Department of Orthopaedic Surgery, Graduate School of Biomedical and Health Sciences, Hiroshima University, Hiroshima, Japan

**Keywords:** Acetabular dysplasia, Hip instability, Bony features

## Abstract

**Purpose:**

To evaluate the morphological and radiographic features of hip instability in hip dysplasia.

**Methods:**

Eighty-four patients who had ultrasonography for the assessment of hip instability and computed tomography scan for the assessment of bony morphology were included. The lateral center–edge angle (LCEA), vertical-center-anterior angle (VCA), acetabular roof obliquity (ARO), acetabular head index (AHI), and acetabular version angle (AVA) were calculated as pelvic parameters. Neck shaft angle (NSA), *α*-angle, femoral offset (FO), and femoral anteversion (FA) were obtained as femoral parameters. The combined anteversion angle (CAA) was defined as the sum of AVA and FA.

**Results:**

Pelvic morphology analysis revealed that LCEA, VCA, and AHI were significantly lower, whereas ARO and AVA were significantly higher in the hip instability group. Furthermore, NSA, FO, and CAA were significantly higher in the hip instability group. The cutoff values for LCEA, VCA, and AHI were 17.6°, 34.7°, and 73.6%, respectively. Multivariate analysis revealed that LCEA and VCA were significantly associated with hip instability, with odds ratios of 1.57 and 1.56, respectively. Hip instabilities were associated with lateral/anterior/superior coverage deficiencies in the pelvis and with the NSA, FO, and CAA in the femur. Furthermore, a correlation between pelvic and femoral morphological parameters suggests that hip instability evaluations should include the evaluation of the anterior/lateral coverage of the acetabulum and femoral parameters.

**Conclusion:**

Our findings suggest that the LCEA, VCA, and AHI could serve as diagnostic markers for hip instability.

## Introduction

Hip instability has been recognized as a potential cause of non-arthritic hip pain and dysfunction in young patients during the assessment and treatment of hip pathologies [1–3]. The etiology of hip instability is multifactorial and includes hip bony morphology, connective tissue disorders, trauma, and iatrogenic (postsurgical) factors [3]. Among these factors, bony hip morphology, such as acetabular dysplasia, is an important contributor to hip instability. Acetabular dysplasia is mainly associated with increased femoral head translation and excessive acetabular limb loading, resulting in hip pain and secondary hip osteoarthritis (OA) [4, 5]. Several reports have described a broad spectrum of alterations in the hip morphology in patients with acetabular dysplasia. Several authors have reported that the bony morphological features of acetabular dysplasia include decreased anterior and lateral coverage of the femoral head, increased acetabular roof obliquity (ARO), and deformities of the proximal femur [6–9]. However, the morphological and radiographic factors predisposing patients to hip instability remain unknown; therefore, objective criteria for the diagnosis of hip instability are needed.

Radiographs are typically used in standard practice for the assessment of bony morphology and associated hip instability. Several radiographic measurements, such as the lateral center–edge angle (LCEA) of Wiberg [10], ARO, and measurement of the vertical-center-anterior angle (VCA) on the false profile view of Lequesne (FPV) [11] have historically been used to determine the morphological features of acetabular dysplasia leading to hip instability, although their limitations to display three-dimensional structures may compromise our ability to correctly diagnose conditions of the hip instability. Other radiographic findings of hip instability, such as femoral head subluxation and broken Shenton’s line [12], have also been reported; however, these findings cannot be detected without OA progression, and patients may have hip instability even if they are radiographically normal.

This study focused on evaluating the relationships between computed tomography (CT)-derived measurements of acetabular dysplasia using CT-based simulation software (ZedHip Lexi Co., Ltd., Tokyo, Japan) [13, 14], including acetabular and femoral morphology, and hip instability. Furthermore, the study aimed to investigate potential cutoff values for radiographic measurements suggesting hip instability in patients with acetabular dysplasia.

## Materials and methods

### Patients

This study was approved by our Institutional Review Board. This study retrospectively reviewed 84 patients (84 hips; 12 males, 72 females, mean age: 46.4 years old, mean body mass index (BMI): 22.3 kg/m^2^) with hip OA due to acetabular dysplasia who consulted our hospital because of hip pain. Acetabular dysplasia was defined as an LCEA < 20º, based on anteroposterior radiograph measurements, and a VCA of < 20º, based on FPV radiograph measurements. The inclusion criteria for this study were patients with grade 1 OA according to the Tönnis classification [15] and grade 1 hip displacement according to the Crowe classification [16]. We excluded patients with a deformed femoral head and those who had previously undergone surgery, which made it difficult to measure bone morphology and hip instability due to bony abnormalities. All patients underwent ultrasonography (US) to assess hip instability and CT. CT scans were obtained from the anterior superior iliac spine (ASIS) to the knee joint through the distal femoral condyles using a 320-row multidetector helical CT scanner (Aquilion ONE, Toshiba Medical Healthcare, Tochigi, Japan) (detector configuration: 80 × 0.5, beam collimation: 40 mm) with a reconstructed slice width of 1.00 mm and a slice interval of 1.00 mm. The CT data were transferred to a CT-based simulation software (ZedHip Lexi Co., Ltd., Tokyo, Japan) to evaluate hip bony morphology [13, 14].

### Assessment of hip instability: US

SONIMAGE HS1/HS1 PRO and SNiBLE diagnostic ultrasound systems and a convex probe (3–13 MHz) (KONICA MINOLTA, Tokyo, Japan) were used [18]. This system and probe clearly showed the femoral head center and acetabulum, even in patients with thick muscles and subcutaneous fat. All patients underwent dynamic US evaluation of the hip joint by two orthopedic surgeons. The patient was instructed to press their lower back flat against the examination table to promote lumbar and pelvic stabilization and to minimize variability in pelvic tilt. Subsequently, each hip was visualized in two positions: For the first position, the patient was placed in the supine position with both hips extended with 0° of abduction and internal rotation, designated as the neutral position; for the second position, the patient was placed in the supine position, and the hip was held in the ‘Patrick position’ with hip abduction and external rotation and the contralateral hip in a neutral position. In all positions, the probe was slightly angled to the sagittal plane to identify the femoral head and the anterior inferior iliac spine (AIIS). The US examiner scanned the femoral head and AIIS from medial to lateral and located the femoral head and highest point of the AIIS. The distance between the anterior edge of the AIIS and the horizontal line to the sclerotic margin of the femoral head was measured to assess the femoral head position. Using this protocol, the difference between the distance, defined as the femoral head translation distance (FTD), in the neutral and Patrick positions, was calculated to evaluate hip stability; an FTD > 2.0 mm was defined as hip instability, as previously reported [18, 19].

This study involved two separate ultrasound examinations conducted several weeks apart to measure inter- and intra-observer reliability. This protocol tested reliability among the physicians at each time point (interobserver reliability) as well as the liability for each orthopedic surgeon individually over time (intra-observer reliability).

### Assessment of hip bone morphology: CT

**Fig. 1:**
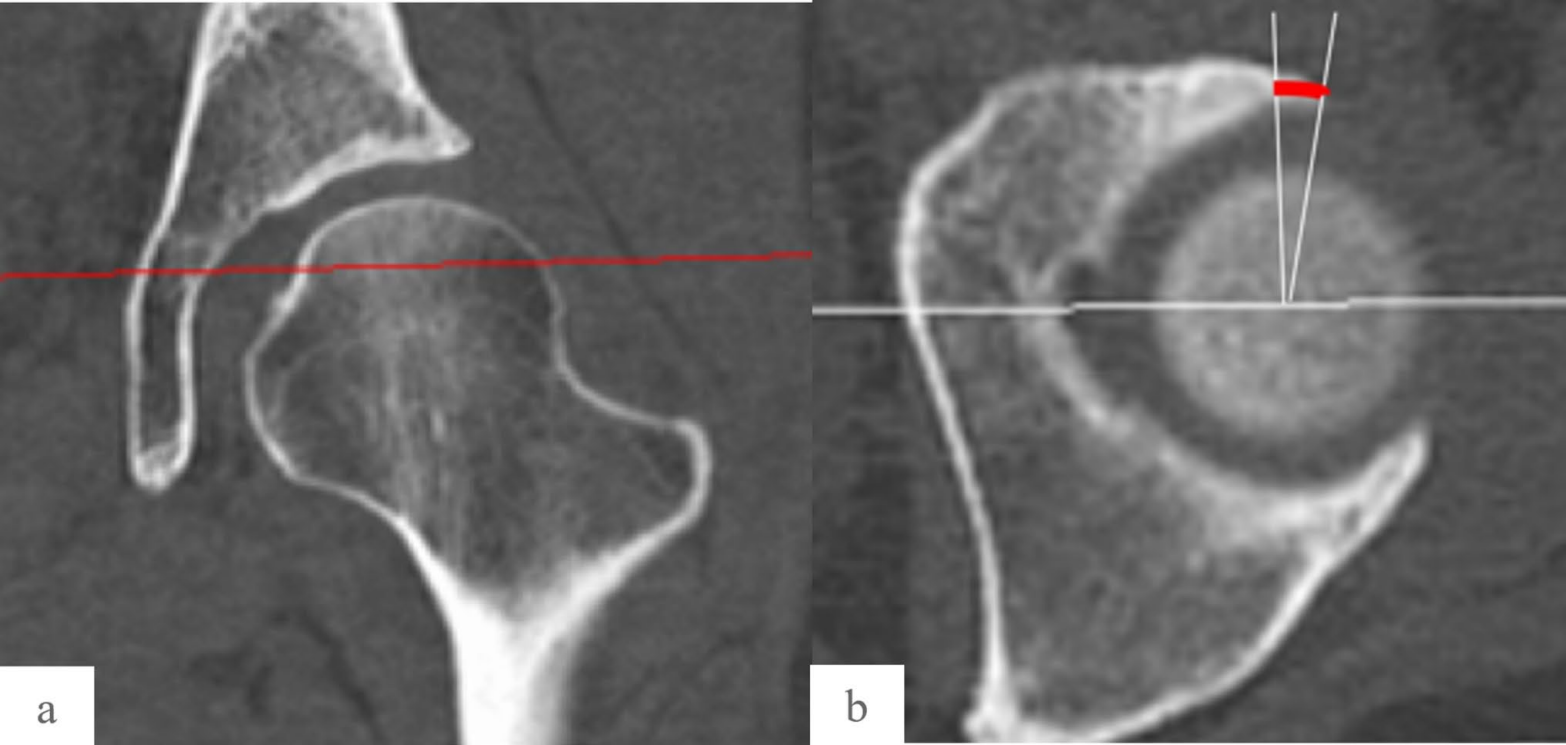
Radiographic indices for evaluating the acetabular version angle. **a** The plane of measurement is the superior quarter of the femoral head in a computed tomography (CT) coronal image obtained at the level of the central section of the femoral head and **b**the acetabular version angle is defined as the angle between a perpendicular line drawn from the center of the femoral head and the anterior rim of the acetabulum on an axial CT image (red curve line)

Patient positioning was standardized with the hips in neutral abduction/adduction and the patellae facing anteriorly. The three-dimensional (3D) CT measurements were calculated using CT-based simulation software (ZedHip Lexi Co., Ltd., Tokyo, Japan) by two orthopedic surgeons [12, 13]. The LCEA, VCA, ARO, acetabular head index (AHI), and acetabular version angle (AVA), obtained at the midpoint of the acetabulum from superior to inferior on axial reconstruction, were calculated on the coronal and sagittal planes, which passed through the center of the femoral head in the functional pelvic plane, as pelvic parameters (Fig. 1). The neck shaft angle (NSA), α-angle, femoral offset (FO) angle, and femoral anteversion (FA) angle were obtained on the femoral coordinate system defined by the International Society of Biomechanics as being the center of the femoral head, the knee center, and both femoral condyles [20]. The FA angle to the transepicondylar axis of the knee was measured as a parameter of the FA on the axial plane. The FO and NSA were measured by rotating the femur by the angle of the FA on the coronal plane, considering the FA as a femoral morphological parameter. The combined anteversion angle (CAA), defined as the sum of the AVA and FA, was also measured.

### Statistical analysis

All data were expressed as mean ± standard deviation (SD), and statistical analysis was performed using the Statcel 3® software (Publisher OMS Ltd., Saitama, Japan). The difference between the two groups for hip stability was determined using Fisher’s exact test. The normal distribution of variables in this study was determined by the Kolmogorov–Smirnov test. Therefore, we determined the differences between the groups for LCEA, VCA, ARO, AHI, NSA, and FO using Student’s t-test, because these variables comprised equality of variance. We determined the differences between the groups for AVA, FA, α-angle, and CAA using Welch’s t-test, because these variables did not comprise equality of variance. The correlation between the morphological parameters and the distance of anterior femoral head translation on US was assessed using Pearson’s correlation coefficient. Receiver operating characteristic (ROC) curve analysis was used to evaluate the quality of instability prediction using CT measurements, with the area under the curve (AUC) as a measure of accuracy and to determine an optimal cutoff value. A multivariate analysis of the risk factors for hip instability was performed using a logistic regression model for variables with *p* < 0.05, including the morphological parameters. Statistical significance was set at *p* < 0.05. The necessary number of patients to identify the difference in each parameter as a primary endpoint, with the assumed delta under the power of 80%, was calculated using G*power 3.1.9.4 [21].

## Results

**Fig. 2:**
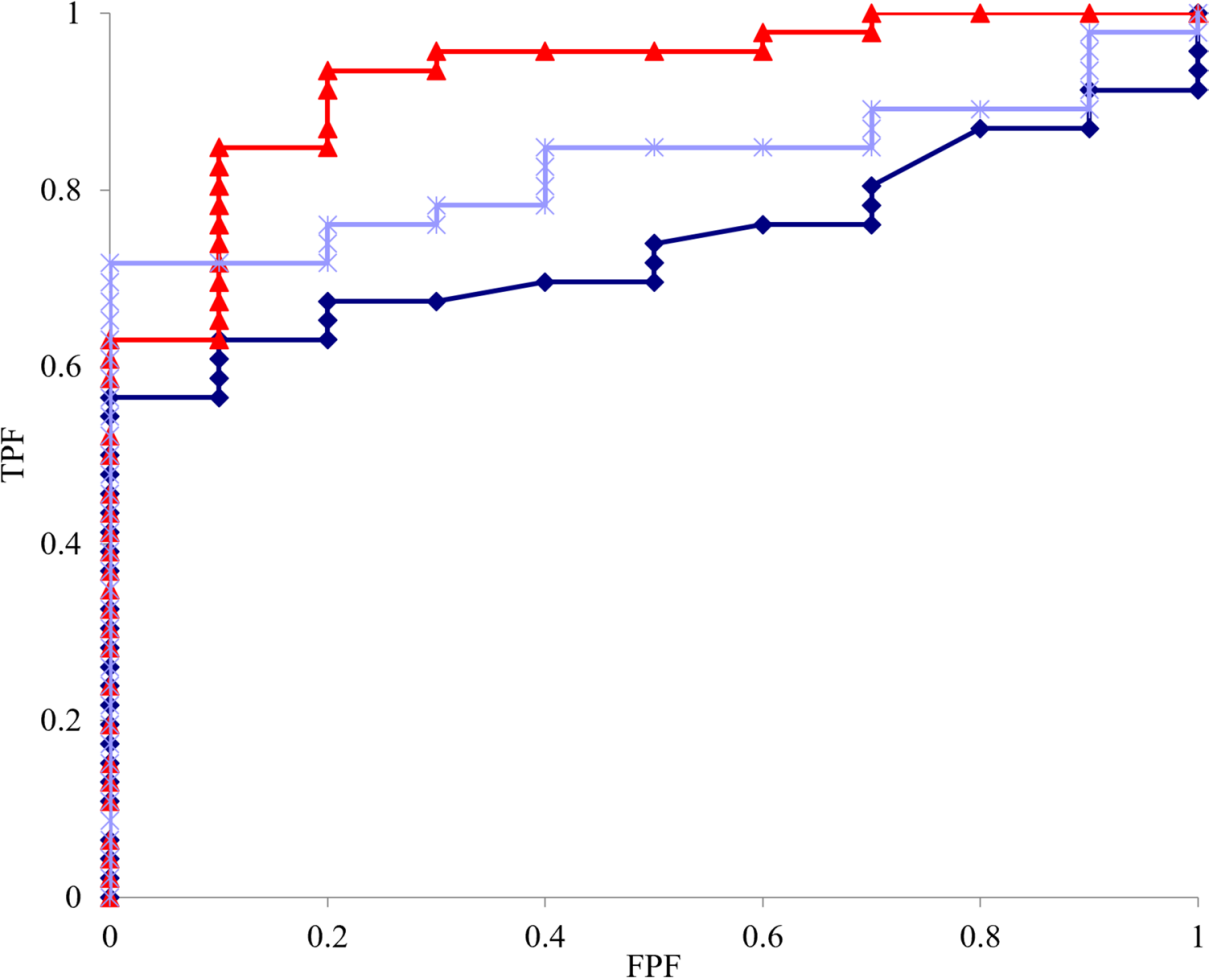
Receiver operating characteristic (ROC) curve analysis between hip instability and the lateral center–edge angle (LCEA), vertical-center-anterior margin angle (VCA), and acetabular head index (AHI). The triangles indicate the VCA. The asterisk indicates the AHI. The diamond line indicates LCEA. TPF: true-positive rate; FPF: false-positive rate

Based on the US evaluation of hip instability, 68 hips were classified as the hip instability group with an FTD of 2.0 mm or more, while 16 hips were classified as the hip stability group with an FTD of < 2.0 mm. The mean FTD was significantly higher in the hip instability group (3.5 ± 0.9 mm) compared to the hip stability group (1.6 ± 0.3 mm). The mean intra- and interobserver ICC were 0.96 (95% CI 0.85–0.99) and 0.91 (95% CI 0.66–0.98) respectively for the analysis of FTD. The analysis of pelvic morphology revealed that the LCEA, VCA, and AHI were significantly lower in the hip instability group, whereas the ARO and AVA were significantly higher (Table 1). As for the femoral parameters, the NSA and FO were significantly higher in the hip instability group. Furthermore, the CAA was significantly higher in the hip instability group. The correlation analysis between pelvic and femoral morphological parameters and hip instability revealed significant negative correlations between the FTD and LCEA, VCA, and AHI and positive correlations between the FTD and the ARO and AVA in the pelvis (Table 2). In the femur, significant positive correlations were observed between the FTD and the NSA, and a negative correlation was observed between the FTD and FO (Table 3). However, these correlations were stronger in the pelvis than in the femur. Furthermore, a significant positive correlation was observed between the CAA and FTD. ROC analysis revealed that the LCEA, VCA, and AHI provided excellent discrimination of the FTD, with AUCs of 0.74, 0.93, and 0.83, respectively (*p* < 0.001), the optimal cutoff values for the CEA, VCA, and AHI were 17.6°, 34.7°, and 73.6% (Fig. 2). Furthermore, logistic regression analysis showed that the LCEA and VCA were independent predictors of hip instability (*p* < 0.05) (Table 4). Correlation analysis revealed that the LCEA was negatively correlated with both the NSA and FO. The VCA also showed a negative correlation with the NSA (Table 5). Moreover, there was a significant positive correlation between the LCEA and VCA and a negative correlation between the LCEA and the ARO and AVA (Table 6).

**Table 1:** Pelvic and femoral morphological parameters in hip instability ± group

		Total	Instability ( +)	Instability (−)	*P*-value
FTD			3.5 (0.9)	1.6 (0.3)	< 0.05
Pelvis					
LCEA	(°)	13.6 (4.9)	12.2 (6.6)	17.9 (1.8)	< 0.05
VCA	(°)	31.3 (7.8)	30.0 (7.4)	39.7 (5.8)	< 0.001
ARO	(°)	19.2 (7.3)	20.2 (6.9)	9.9 (2.9)	< 0.05
AVA	(°)	25.7 (10.6)	26.8 (10.5)	15.7 (7.0)	< 0.05
AHI	(%)	69.4 (7.2)	68.6 (7.4)	74.6 (2.0)	< 0.05
Femoral head					
FA	(°)	25.8 (10.7)	26.7 (10.4)	19.6 (10.9)	0.07
NSA	(°)	135.1 (6.4)	136.1 (6.1)	128.3 (2.7)	< 0.001
α-angle	(°)	51.7 (11.8)	51.5 (12.0)	52.9 (10.9)	0.43
FO	(mm)	27.5 (5.9)	26.9 (5.9)	31.5 (4.0)	< 0.05
CAA					
	(°)	43.9 (11.4)	45.6 (10.7)	28.7 (5.1)	< 0.001

LCEA, lateral center–edge angle; VCA, vertical-center-anterior angle; ARO, acetabular roof obliquity; AVA, acetabular version angle; AHI, acetabular head index; FA, femoral anteversion; NSA, neck shaft angle; FO, femoral offset; CAA, combined anteversion angle

**Table 2:** Correlation between pelvic parameters and hip instability

		LCEA	VCA	ARO	AVA	AHI
FTD	r	−0.69	−0.67	0.76	0.54	−0.62
	*p*-value	< 0.001	< 0.001	< 0.001	< 0.001	< 0.001

**Table 3:** Correlation between femoral parameters and hip instability

		FA	NSA	*α* angle	FO	CAA
FTD	r	0.33	0.44	−0.1	−0.43	0.41
	*p*-value	0.16	< 0.001	0.5	< 0.05	< 0.05

FTD, femoral head translation distance; LCEA, lateral center–edge angle; VCA, vertical-center-anterior angle; ARO, acetabular roof obliquity; AVA, acetabular version angle; AHI, acetabular head index; FA, femoral anteversion; NSA, neck shaft angle; FO, femoral offset; CAA, combined anteversion angle

**Table 4:** Multivariate analysis of the factors influencing hip instability

	Odds ratio	95%CI	*P*-values
LCEA	1.57	−0.36–1.24	0.03
VCA	1.56	0.01–1.12	0.03

LCEA, lateral center–edge angle; VCA, vertical-center-anterior angle

**Table 5:** Correlation among pelvic and femoral bone morphology

		NSA	FA	FO
LCEA	r	−0.57	0.16	−0.38
	*p*-value	< 0.001	0.84	< 0.05
VCA	r	−0.69	−0.15	0.33
	*p*-value	< 0.001	0.43	< 0.05

**Table 6:** Correlation among pelvic bone morphology

		VCA	ARO	AVA
LCEA	r	0.44	−0.50	−0.34
	*p*-value	< 0.001	< 0.001	< 0.05

## Discussion

An accurate evaluation of hip instability is critical for the diagnosis and successful treatment of acetabular dysplasia. Patients with acetabular dysplasia not only have acetabular morphological abnormalities, but also abnormal proximal femoral morphology, which is also associated with hip instability, although little information is available regarding the relationship between morphological features and hip instability. In this study, several possible relationships were identified between morphological features, including femoral bony morphology and hip instability, which need to be clinically investigated.

Acetabular dysplasia is associated with morphological abnormalities ranging from acetabular to femoral bone morphology. Regarding acetabular morphological features, patients with acetabular dysplasia have shallow hip sockets and a short distance between the bilateral AIIS and the low-angle iliac wing on the axial and sagittal planes, which leads to hip instability [22, 23]. Patients with acetabular dysplasia also have proximal femoral abnormalities, such as a high neck shaft angle [24], whereas little is known about the relationship between hip instability and femoral bony abnormalities. Because both morphological features are associated with hip instability, there is a need to identify objective imaging findings associated with hip instability, as history and physical examination may be unreliable [25, 26]. Several radiographic measurements have been proposed to determine whether painful symptoms are caused by hip instability; however, 2D parameters cannot accurately describe its 3D structure [27–29]. Furthermore, not only femoral bone morphology, but also its relationship with acetabular bone morphology affects hip instability [29]; thus, a 3D structural evaluation of acetabular and femoral bone morphology and its relationship is inevitable [30, 31].

In this series, approximately 81% of the patients with acetabular dysplasia exhibited hip instability. These instabilities were associated with lateral, anterior, and superior coverage deficiencies in the pelvis and with the NSA and AHI in the femur and CAA. The optimal cutoff values for the CEA, VCA, and AHI were 17.6°, 34.7°, and 73.6%, respectively. The outcomes of our research are in accordance with a prior study conducted by Murphy et al. who posited a negative correlation between prognosis and bone morphology in cases exhibiting an LCEA of < 16° [32]. Furthermore, a correlation between pelvic and femoral morphological parameters suggests that the evaluation of hip instability should not be limited to a 2D assessment of the CEA but should also include an evaluation of the anterior/lateral coverage of the acetabulum and femoral parameters. As previously reported associations between proximal femoral abnormalities and hip biomechanics in hip dysplasia [33], our findings suggest that these parameters could serve as new diagnostic markers and inform treatment strategies for acetabular dysplasia, such as rehabilitation and pelvic osteotomy.

However, the study has some limitations, such as its retrospective nature, lack of a control arm, a fairly small number of patients, use of computer-simulated scenarios (instead of in vivo tests), and the fact that bony morphology alone is not sufficient to determine whether a hip is unstable. Moreover, the study population consisted of a cohort of a single surgeon’s patients with acetabular dysplasia, and the diagnosis of hip instability was a US evaluation.

Despite these potential limitations, the main question of the study is very valid: Basing our surgical decision-making process on poor imaging may lead to an over- or underestimation of the procedures required by the patient. As a result, hip instability is a clinical diagnosis that radiologists should be aware of, and our findings indicated that instabilities were associated with lateral, anterior, and superior coverage deficiencies in the pelvis and with the NSA, LCEA, AHI, and CAA in the femur. Furthermore, a correlation between pelvic and femoral morphological parameters suggested that the evaluation of hip instability should not be limited to a 2D assessment of the LCEA but should also include an evaluation of the anterior/lateral coverage of the acetabulum and femoral parameters. Our findings suggest that these parameters could serve as new diagnostic markers and inform treatment strategies for acetabular dysplasia, such as rehabilitation and pelvic osteotomy.

## Data Availability

No datasets were generated or analyzed during the current study.
